# Mechanical Wounding Induces Rapid RNA-Degrading Activity Mediated by the S-like Ribonuclease PvRNS2 in Common Bean

**DOI:** 10.3390/plants15121907

**Published:** 2026-06-19

**Authors:** Lucia O. Pareja, Gregorio Galvez-Valdivieso, Pedro Piedras, Mercedes Diaz-Baena

**Affiliations:** Grupo de Fisiología Molecular y Biotecnología de Plantas, Departamento de Botánica, Ecología y Fisiología Vegetal, Campus de Rabanales, Edif. Severo Ochoa, Universidad de Córdoba, 14071 Córdoba, Spain; b82orpal@uco.es (L.O.P.); b32gavag@uco.es (G.G.-V.); b42dibam@uco.es (M.D.-B.)

**Keywords:** ribonuclease T2, wounding, common bean, nucleotides, RNA

## Abstract

Common bean (*Phaseolus vulgaris*) is an important crop for human nutrition due to its high protein content and capacity to fix atmospheric nitrogen. However, crop productivity is frequently compromised by biotic and abiotic stresses, among which wounding represents a highly prevalent challenge. Thus, understanding early molecular and biochemical responses to tissue damage is essential for improving plant stress resilience. We have investigated the effects of mechanical wounding on nucleic acid-degrading activities in the common bean. Mechanical wounding of leaves rapidly induced ribonuclease activity, whereas nuclease activities remained unchanged. Gel activity assays revealed a predominant ribonuclease, which was identified by proteomic analysis as PvRNS2, a member of the S-like RNase T2 family. This wound-induced ribonuclease was inhibited more strongly by nucleoside di- and triphosphate than by the corresponding nucleoside monophosphate. The increase in ribonuclease activity correlated with a rapid and transient induction of *PvRNS2* expression, which peaked at 2 h after injury (600-fold increase). A similar transcriptional response was observed in radicles subjected to mechanical damage (55-fold increase), indicating that *PvRNS2* responds to wounding in both aerial and subterranean tissues. In contrast, the wound-induced increase in *PvRNS2* expression was not associated with a coordinated upregulation of genes encoding enzymes involved in downstream nucleotide degradation. Together, these results identify PvRNS2 as a major contributor to wound-induced RNA turnover in the common bean and support the involvement of RNA metabolism in early responses to mechanical damage. The participation of ribonucleases in the wound response of economically vital legumes remains unexplored. This work addresses this knowledge gap, establishing a new framework for understanding nucleic acid degradation during legume defense.

## 1. Introduction

Plants are sessile organisms and, therefore, must continuously adjust their growth and development in response to changes in their environment to maintain their homeostasis. Wounding is an environmental stress to which plants are frequently exposed. It can arise from abiotic factors such as weather conditions, as well as from biotic interactions, including pathogens and herbivores. Tissue damage caused by wounding leads to dysfunctions in the plasma membrane and cell wall [1,2]. Wounding is a major factor negatively affecting plant fitness and productivity, as it causes loss of nutrients and entry of pathogens. In response to wounding, plants activate complex defense pathways to prevent the advance of opportunistic pathogens and to regenerate the damaged tissues through healing [3]. Research on these pathways has focused on identifying molecular and cellular events underlying these defense and regeneration mechanisms [4], as well as on the establishment of immune response through the perception of damage-associated molecular patterns (DAMPs) [5]. DAMPs can be classified into two groups: constitutive or primary DAMPs, which correspond to pre-existing cellular compounds that diffuse into the apoplast from damaged cells, and inducible or secondary DAMPs, which are synthesized by the plant upon perceiving damage [5]. Constitutive or primary DAMPs include ATP, NAD(P), glutamate, DNA, and cell wall fragments, whereas inducible or secondary DAMPs include constitutively expressed proteins that are cleaved upon tissue injury [5,6].

The role of DNA as DAMP has gained much attention in recent years [7,8,9,10]. However, the mechanism by which extracellular self-DNA triggers plant response is poorly understood. Although some evidence has been reported for the existence of DNA receptors in plants, no DNA receptors, similar to those found in animals, have been identified so far in plants [11]. In addition to DNA, cell damage also results in the release of RNA, a nucleic acid more abundant than DNA in plant cells, with rRNA representing the larger fraction among RNAs [12]. In plants, the role of RNA as DAMP has received little attention. Recently, it has been demonstrated that pepper leaves preinfiltrated with self-eRNA activate immunity against viral and bacterial pathogens [13].

Ribonucleases are essential enzymes that catalyze the degradation of RNA. Among them, the ribonuclease T2 family is the most widely distributed among living beings, with members identified in both bacteria and eukaryotes [14]. In every eukaryotic genome that has been sequenced to date, at least one gene from this family has been found, thus underscoring the fundamental biological role of this enzyme family. RNAses T2 are mainly associated with the secretory pathway and have been implicated in the maintenance of cellular homeostasis, fulfilling diverse roles from stress responses to control of self-pollen rejection [15]. These enzymes are endoribonucleases capable of cleaving RNA after all four nucleobases and mainly display an acidic pH optimum [14]. The crystal structure of the human RNase T2 [16] revealed the existence of four disulfide bridges essential for maintaining its structural integrity. The cysteines involved in these four disulfide bridges are conserved in most plant RNases T2 [17]. The proposed functions for this family of ribonucleases include housekeeping roles, production of t-RNA-derived sRNAs, defense activities, and gametophytic self-incompatibility [15]. S-like Ribonucleases T2 in common bean are composed of four members [17], which have previously been associated with situations involving high nutrient mobilization, including salt stress [17], germination and seedling development [18,19], and seed development in the mother plant [20].

Common bean (*Phaseolus vulgaris* L.) is the most important legume for direct human consumption. Its high protein, dietary fiber, carbohydrate, and mineral content make it a key component in healthy diets [21]. In addition to its nutritional value, common bean cultivation offers significant environmental benefits. Common beans, like other legumes, can fix nitrogen through symbiotic relationships with bacteria, reducing the need for nitrogen fertilizers and thereby lowering the associated carbon footprint. Increased production and consumption of common beans can therefore contribute to sustainable agriculture and climate change mitigation [21]. Here, we employ mechanical wounding as an approach to investigate the molecular responses to wounding in common bean. This strategy has been widely used to generate reproducible damage-associated signaling in plant tissues [22,23,24,25,26]. Whether ribonucleases T2 participate in wound-induced responses, potentially contributing to nutrient salvage or defense signaling following tissue damage in common bean, has not yet been addressed. We show that mechanical wounding induces an upregulation of several S-like ribonuclease T2 genes followed by increased ribonuclease activity both in leaves and in radicles of common bean. Furthermore, we characterize the RNase T2 gene that responds most rapidly to wounding, and we propose that the protein encoded by this gene is responsible for the high ribonuclease activity induced by wounding. To date, the specific role of these ribonucleases during wounding in high-value legumes has not been investigated. This study addresses this oversight, offering unprecedented insights into how nucleic acid degradation contributes to the defensive repertoire of legumes.

## 2. Results

### 2.1. Wounding Induces Ribonuclease Activity in Common Bean Leaves

**Figure 1:**
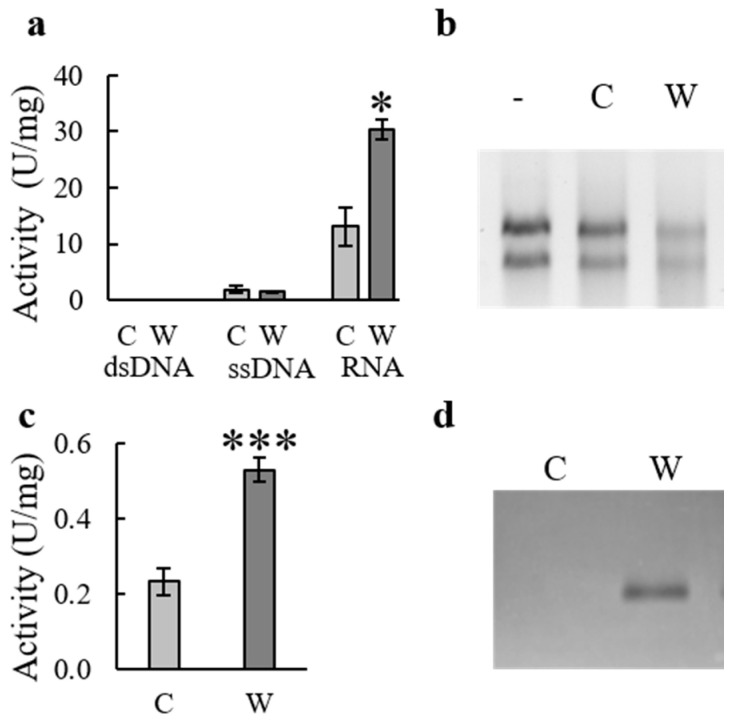
Nucleic acid-degrading activities in wounded leaves. Nucleic acid-degrading activity was determined in crude extracts from leaves collected 2 h after wounding (W) and from leaves not treated as a control (C). (**a**) In vitro assay using as substrates dsDNA, ssDNA, or RNA. The activities are the mean ± SE of three biological replicates. (**b**) Ribonuclease activity assayed with rRNA as substrate using the agarose gel assay. Lane -: reaction mixture without crude extracts; Lane C: reaction mixture with crude extracts from untreated leaves; Lane W: reaction mixture with crude extract from wounded leaves. (**c**) In vitro ribonuclease assay using tRNA as substrate. The activities are the mean ± SE of three biological replicates. (**d**) In-gel ribonuclease assay, performed with crude extracts from not treated (C) or wounded leaves (W). A *t*-test was performed for in vitro assays between values for non-treated and wounded leaves. A significant *p* value is indicated using asterisks (* *p* < 0.05 and *** *p* < 0.001).

**Figure 2:**
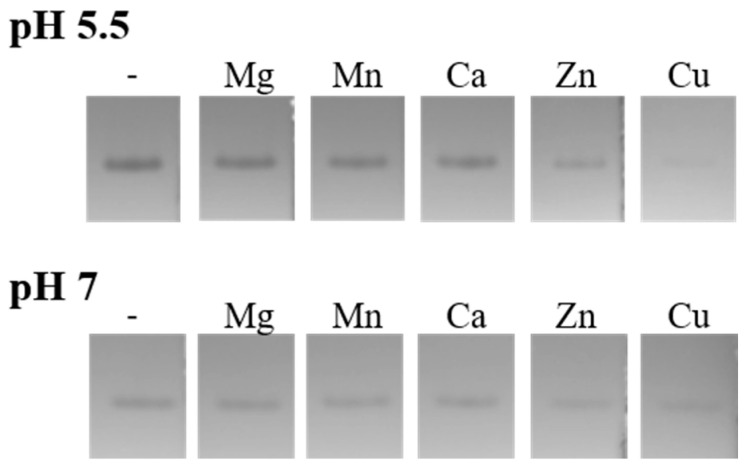
Effect of cations on wounding-induced ribonuclease in common bean leaves at acidic and neutral pH. In-gel ribonuclease activity was assayed in the absence (-) or presence of the indicated cations at a final concentration of 2 mM under acidic and neutral pH conditions in crude extracts obtained from common bean leaves 2 h after wounding. 2 μg of total protein was loaded per lane.

The effect of mechanical wounding on nucleic acid-degrading activities was determined in crude extracts obtained from common bean leaves (Figure 1). The in vitro activity assay showed an increase in activity only with RNA as substrate (Figure 1a). The activity with dsDNA was undetectable, and no changes were observed with ssDNA as substrate (Figure 1a). The induction of RNase activity was also obtained with rRNA (Figure 1b) and tRNA (Figure 1c) as substrates, indicating broad RNA substrate specificity. Ribonuclease activity was further analyzed by in-gel assay to determine the number of ribonucleases present in the extracts. A major activity band with an apparent molecular mass of approximately 17 kDa was detected exclusively in extracts from wounded leaves (Figure 1d). To further characterize this activity, in-gel assays were performed in the presence of different divalent cations at both acidic and neutral pH. Under all conditions tested, only the 17 kDa ribonuclease activity was detected, and no additional activities were observed (Figure 2). Notably, ribonuclease activity was inhibited in the presence of Zn^2+^ and Cu^2+^, whereas other cations had no apparent effect (Figure 2).

**Figure 3:**
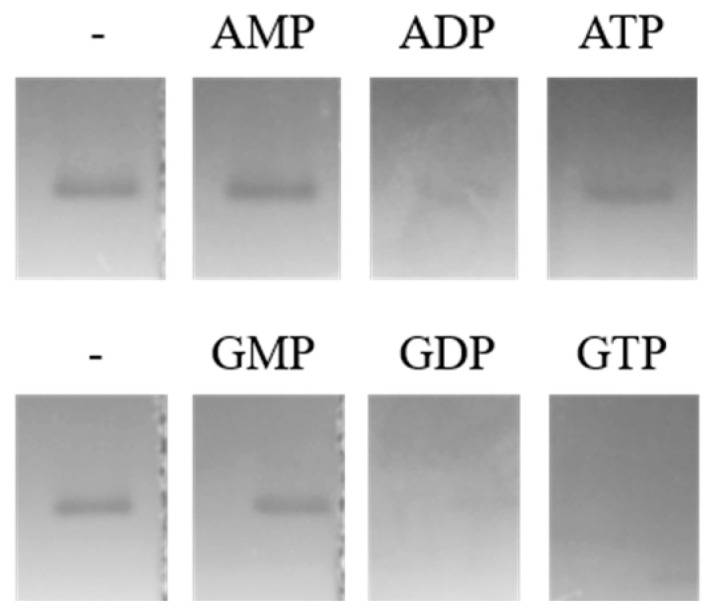
Effect of adenosine and guanosine nucleotides on wounding-induced ribonuclease. In-gel ribonuclease assay was performed in the absence (-) or presence of the indicated nucleotides at the final concentration of 1 mM in crude extracts obtained from common bean leaves 2 h after wounding. 2 μg of total protein was loaded per lane.

The effect of cellular energy status on ribonuclease activity was assessed by analyzing its activity in the presence of nucleoside monophosphate, diphosphate, and triphosphate of adenosine and guanosine (Figure 3). RNase activity was inhibited by the presence of nucleoside diphosphate and triphosphate, and a weaker inhibitory effect was observed for nucleoside monophosphate. The greatest inhibition was observed with guanosine nucleotide, particularly GDP and GTP (Figure 3).

### 2.2. Identification of the Wounding-Induced Ribonuclease

To identify the ribonuclease responsible for the detected activity, the portion of the gel containing the 17 kDa activity was excised and subjected to proteomic analysis. The only ribonuclease identified in this fraction was PvRNS2 (Table S1), a previously identified S-like ribonuclease T2 from common bean [17]. The cDNA-encoding PvRNS2 was obtained from RNA isolated from wounded leaves 2 h after wounding and sequenced. Sequence analysis revealed the presence of a signal peptide that was not annotated in the corresponding PvRNS2 entry in the Phytozome database. The deduced amino acid sequence predicted that PvRNS2 has a molecular mass of 25.2 kDa, including the signal peptide and 22.7 kDa after its removal. PvRNS2 is predicted to be a soluble protein with an apoplastic location. This molecular mass (22.7 kDa) is noticeably higher than the migrated activity (17 kDa), which can be explained by the in-gel assay being performed under non-reducing conditions (SDS-PAGE without DTT).

### 2.3. Expression of S-like Ribonuclease T2 in Wounded Common Bean Leaves

**Figure 4:**
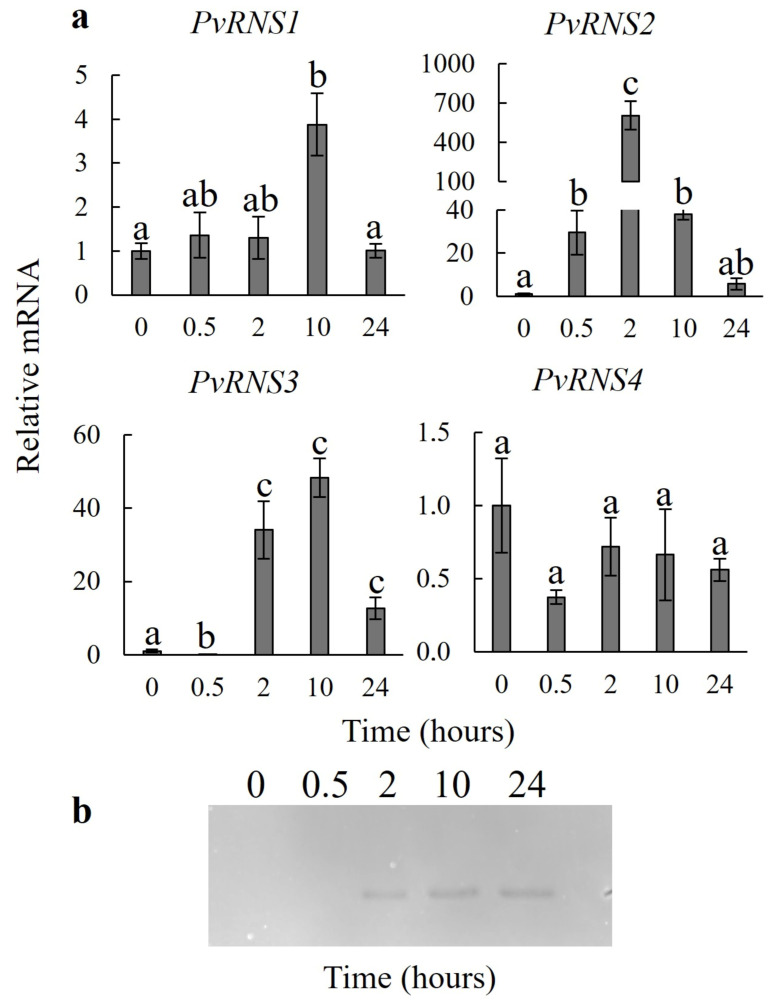
Expression pattern of S-like ribonuclease T2 and ribonuclease activity in wounded leaves at different times after wounding. (**a**) S-like ribonuclease T2 expression analysis was performed using qRT-PCR on total RNA samples extracted from leaves of the common bean at the indicated time after wounding. The relative expression level was normalized using the geometric mean of two reference genes and analyzed using the 2^−ΔCT^ method. Values are the mean ± SE of four biological replicates with three technical replicates for each. Statistical analyses were performed on log10-transformed data. Different letters indicate significant differences among treatments according to Tukey’s HSD or Games–Howell post hoc tests, as appropriate, *p* < 0.05. (**b**) In-gel ribonuclease activity was determined in acetate buffer pH 5.5 using crude extracts obtained from leaves at the indicated time after wounding. Equal amounts of total protein (2 μg) were loaded per lane.

The expression profile of the four genes constituting the S-like RNase T2 was analyzed at different times after wounding the leaves (Figure 4a). Differential behavior was observed among the four analyzed genes. *PvRNS4* did not exhibit significant changes in transcript abundance at any analyzed time point. The relative expression of the *PvRNS1* gene increased significantly in relation to non-wounded leaves only 10 h after wounding. In contrast, *PvRNS2* and *PvRNS3* showed significant induction after wounding, although with distinct temporal patterns. *PvRNS2* expression increased rapidly, with significant upregulation detected as early as 30 min after wounding, reaching a maximum at 2 h post-wounding. In contrast, *PvRNS3* did not show significant changes at 30 min, and the peak of expression was not observed at 2 h, whereas differences in expression were observed compared to untreated leaves at 2, 10, and 24 h after wounding (Figure 4a). *PvRNS2* was the gene whose expression increased the most relative to the control, with a 700-fold increase at 2 h compared to the expression in untreated leaves.

Ribonuclease activity after wounding was analyzed by in-gel assay over the same time course (Figure 4b). At all of the time points analyzed, only the 17 kDa ribonuclease activity was detected. This activity was determined 2 h after wounding, being higher in leaves at 10 and 24 h after wounding (Figure 4b). The fact that the expression of the *PvRNS2* gene is the one that relatively increases the most in response to injury would support the fact that the only activity determined in the gel is the 17 kDa band identified as PvRNS2.

### 2.4. S-like Ribonuclease T2 Gene Expression and Ribonuclease Activity in Radicles in Response to Wounding and Methyl Jasmonate

**Figure 5:**
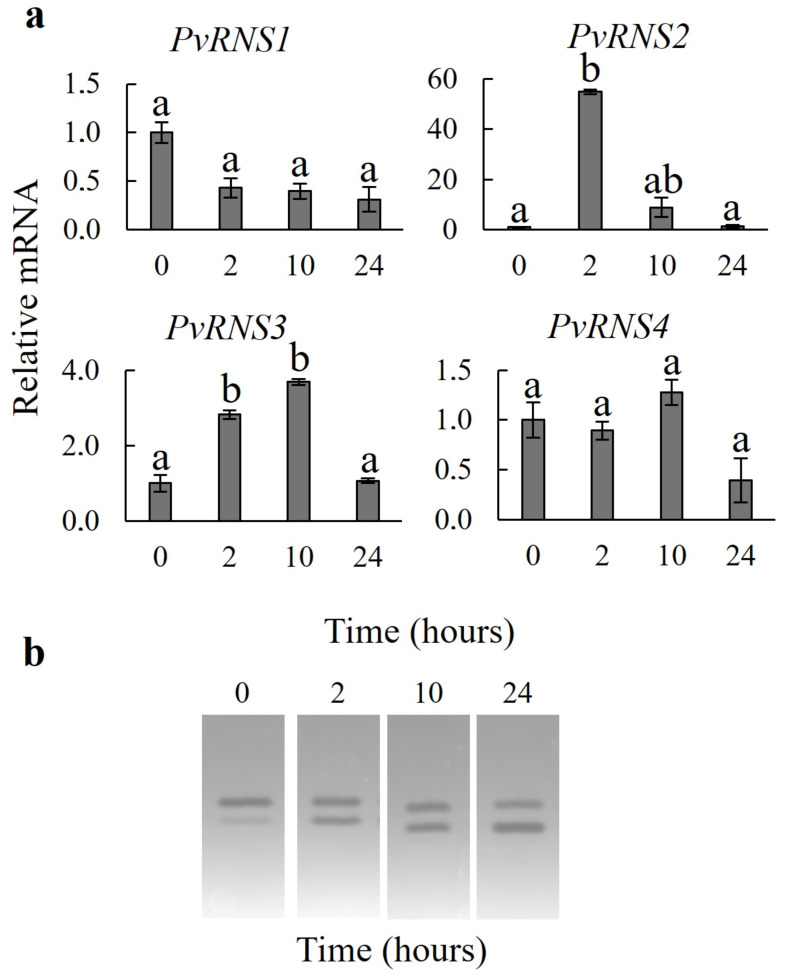
Expression pattern of S-like ribonuclease T2 and ribonuclease activity in wounded radicles at different times after wounding. (**a**) S-like ribonuclease T2 expression analysis was performed using qRT-PCR on total RNA samples extracted from radicles of common bean at the indicated time after wounding. The relative expression level was normalized using the geometric mean of two reference genes and analyzed using the 2^−ΔΔCT^ method. Values are the mean ± SE of four biological replicates with three technical replicates for each. Statistical analyses were performed on log10-transformed data. Different letters indicate significant differences among treatments according to Tukey’s HSD or Games–Howell post hoc tests, as appropriate, *p* < 0.05. (**b**) In-gel ribonuclease activity was determined in acetate buffer pH 5.5 using crude extracts obtained from radicles at the indicated time after wounding. Equal amounts of total protein (4 μg) were loaded per lane.

To determine whether the observed induction in *PvRNS2* expression and ribonuclease activity was specific to wounded leaves or represented a more generally occurring response, S-like RNase T2 expression was analyzed in radicles following mechanical wounding at 2, 10, and 24 h (Figure 5a). The *PvRNS1* and *PvRNS4* genes did not show significant changes in their expression at any of the times analyzed after mechanical damage. *PvRNS2* and *PvRNS3* expression were also rapidly and transiently induced after wounding in radicles from common bean, with increased transcript levels detected 2 and 10 h after wounding, but no significant differences were observed at 24 h (Figure 5a). As in leaves, the peak expression for *PvRNS2* (2 h) was earlier than that observed for *PvRNS3* (10 h), and the highest induction in expression was observed for *PvRNS2* (50-fold increase).

In the gel activity assay on radicles, two proteins with activity and apparent mobility of 16 and 17 kDa were determined. The 16 kDa activity was not modified in wounded radicles, while the 17 kDa activity increased in radicles at all time points analyzed (Figure 5b), as occurred in leaves.

**Figure 6:**
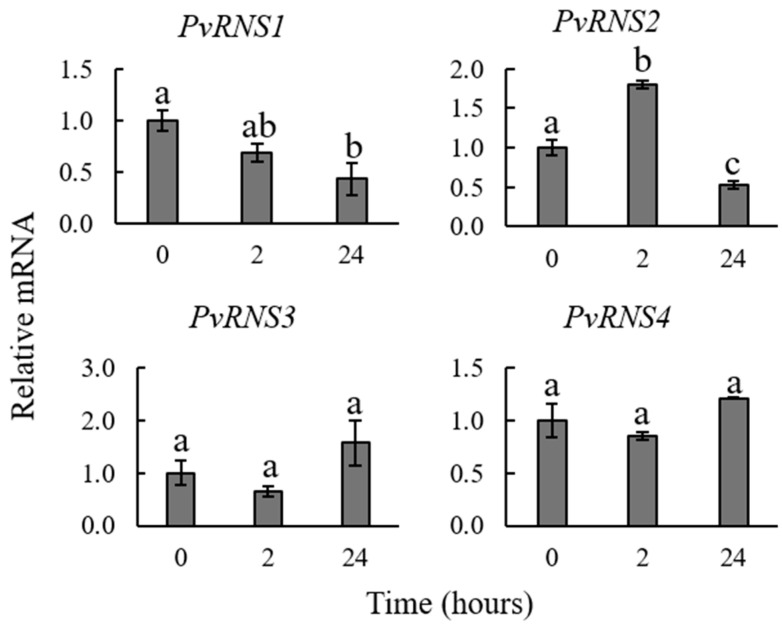
Expression pattern of S-like ribonuclease T2 in radicles after treatment with MeJA. S-like ribonuclease T2 expression analysis was performed using qRT-PCR on total RNA samples extracted from radicles of common bean at the indicated time after MeJA treatment. The relative expression level was normalized using the geometric mean of two reference genes and analyzed using the 2^−ΔΔCT^ method. Values are the mean ± SE of four biological replicates with three technical replicates for each. Statistical analyses were performed on log10-transformed data. Different letters indicate significant differences among treatments according to Tukey’s HSD or Games–Howell post hoc tests, as appropriate, *p* < 0.05.

The expression of the four S-like ribonuclease T2 genes was also analyzed in radicles of seedlings treated with methyl jasmonate (MeJA), a hormone associated with wound signaling. Among the four genes analyzed, only *PvRNS2* showed a significant increase in expression at 2 h after MeJA treatment, whereas no significant changes in expression were detected for any of the genes at 24 h after treatment (Figure 6).

**Figure 7:**
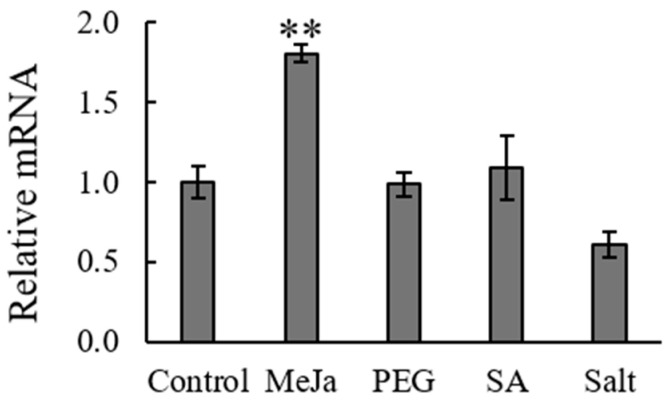
Expression pattern of PvRNS2 in radicles exposed to MeJA, hydric stress (PEG), SA, and salt stress. *PvRNS2* expression analysis was performed using qRT-PCR on total RNA samples extracted from radicles of common bean after 2 h treatment with MeJA (250 μM), PEG 8000 (7.5%), SA (5 mM), and salt (200 mM). The relative expression level was normalized using the geometric mean of two reference genes and analyzed using the 2^−ΔΔCT^ method. Values are the mean ± SE of four biological replicates with three technical replicates for each. Untreated radicles were used as the control. Bars represent mean ± SE. Asterisks indicate significant differences compared with the control according to Student’s *t*-test (** *p* < 0.01).

The changes in expression observed only for *PvRNS2* and in short response to MeJA prompted us to analyze whether the expression of this gene is also modified by other treatments such as water stress induced by PEG, salt stress, or the hormone salicylate (Figure 7). *PvRNS2* expression was only significantly affected by treatment with MeJA (Figure 7).

### 2.5. Effect of Wounding on the Expression of Other Genes Related to Nucleotide Metabolism

**Figure 8:**
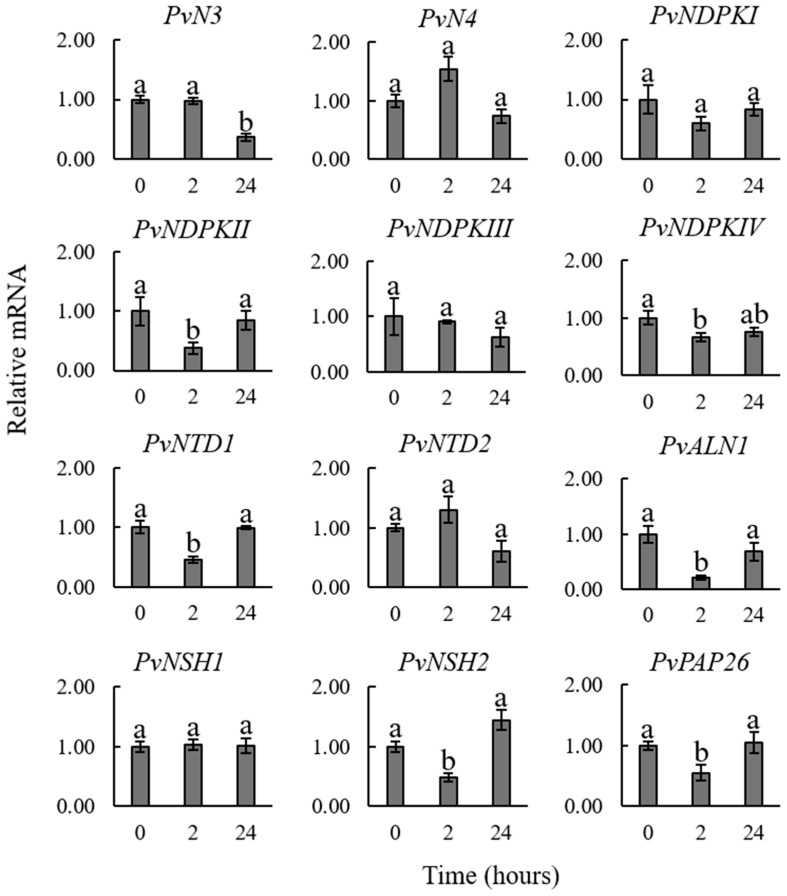
Expression pattern of indicated genes related to nucleotide metabolism in wounded leaves. Expression analysis was performed using qRT-PCR on total RNA samples extracted from leaves of common bean at the indicated time after wounding. The relative expression level was normalized using the geometric mean of two reference genes and analyzed using the 2^−ΔΔCT^ method. Values are the mean ± SE of four biological replicates with three technical replicates for each. Statistical analyses were performed on log10-transformed data. Different letters indicate significant differences among treatments according to Tukey’s HSD or Games–Howell post hoc tests, as appropriate, *p* < 0.05.

The rapid and strong induction of *PvRNS2* suggests the involvement of broader alterations in RNA and nucleotide metabolism during the wound response. Therefore, we decided to analyze the expression of other genes related to nucleotide and nucleic acid metabolism in wounded common bean leaves at 2 and 24 h after wounding (Figure 8). Members of the S1/P1 nucleases, which have been involved in the mobilization of nucleic acids during leaf senescence [27], were examined. Of the five S1/P1 nuclease genes identified in common bean, only *PvN3* and *PvN4* were detected in leaves under the conditions tested, with a decrease in expression observed for *PvN3* at 24 h after wounding, whereas the expression of *PvN4* was not significantly modified by wounding (Figure 8). Nucleoside diphosphate kinases (NDPK), which play a central role in maintaining cellular nucleoside triphosphate pools, were also analyzed. All four NDPK genes (*PvNDPKI* to *IV*) were expressed in bean leaves; however, only *PvNDPKII* and *PvNDPKIV* showed a significant change in expression at 2 h after wounding (Figure 8). Among the putative nucleotidases and the nucleosidases analyzed, transcript levels of *PvNTD1* and *PvNSH2* were significantly reduced after wounding (Figure 8), whereas no significant changes were detected for *PvNTD2* or *PvNSH1*. Finally, the expression of the phosphatase III-B gene, *PvPAP26* [28], was also examined, given that its *Arabidopsis* homolog (*AtPAP26*) has been proposed to function in the degradation of nucleotides in vacuoles downstream of ribonuclease activity [29], and *PvPAP26* transcript levels decreased only at 2 h after wounding (Figure 8). Expression of the gene encoding allantoinase, *PvALN1*, also decreased significantly at 2 h after injury (Figure 8).

## 3. Discussion

Mechanical wounding represents a major threat for plants, as it not only causes the loss of cellular integrity but also facilitates pathogen entry and nutrient leakage. Plants are sessile organisms that have developed sophisticated mechanisms to rapidly perceive tissue damage and activate appropriate defense and repair responses. Mechanical wounding is widely used to simulate herbivore damage and has proven effective for dissecting molecular responses associated with injury and defense signaling [22,25,26,30]. In this study, mechanical wounding of common bean leaves induced a rapid induction of RNA-degrading activity, with no detectable increase in activity toward single- or double-stranded DNA. This specificity suggests that the wound response involves targeted RNA turnover rather than a general activation of nucleases. The functional role of induced ribonuclease activity must be contextualized within the species-specific ecophysiological traits of *Phaseolus vulgaris*, a leguminous plant that obtains its nitrogen by fixing it through nodules in its roots, and with purine nucleotides playing a relevant role in the synthesis of organic nitrogen. Leguminous biological nitrogen fixation within root nodules is an intensely energetic process that imposes an exceptionally high demand for phosphorus. Consequently, the homeostasis of nitrogen and phosphorus is tightly interconnected in this species. Upon mechanical wounding, cellular disruption leads to localized tissue damage. Under these conditions, ribonuclease activity could act as a pivotal enzymatic hub driving nutrient recycling and homeostasis maintenance, allowing the recovery of phosphate and nitrogenous compounds toward healthy tissues or active nitrogen-fixing nodules.

The in-gel activity assay consistently revealed a major ribonuclease activity with an apparent molecular mass of 17 kDa, detectable as early as 2 h after injury and persisting across the analyzed time course, with maximal activity at 24 h. Proteomic analysis identified this activity as PvRNS2, a member of the S-like ribonuclease T2 previously described in common bean [17]. Sequencing of the *PvRNS2* cDNA allowed us to correct the existing database annotation, revealing the presence of a signal peptide and suggesting extracellular localization of the protein. Although the predicted molecular mass of the mature PvRNS2 protein is higher than the calculated value from the in-gel assay, this discrepancy can be explained by the partial denaturation conditions required to preserve enzymatic activity during electrophoresis. PvRNS2 contains the eight putative Cys residues involved in disulfide bridges [17]. The discrepancy between the theoretical molecular weight (22.7 kDa) and the observed mobility under non-reducing SDS-PAGE (17 kDa) strongly supports the functional involvement of all eight cysteine residues in intramolecular disulfide bonds. Without DTT-mediated reduction, these covalent linkages prevent full polypeptide extension by SDS, leaving a rigid, compact core that migrates faster through the polyacrylamide matrix, underestimating its true molecular mass.

The wounding-induced ribonuclease activity showed differential sensitivity to mono-, di-, and triphosphates, with stronger inhibition observed in the presence of adenosine and guanosine di- and tri-phosphates than with their corresponding monophosphates. A similar regulatory pattern was previously described for the salt-induced ribonuclease activity observed in radicles of common bean [17], suggesting that the ribonuclease T2 activity may be finely regulated by cellular energy status or by the cytoplasmic nucleotide pools. High levels of nucleoside di- and triphosphates (NTP/NDP) act as a negative feedback mechanism, inhibiting RNA degradation under energy-sufficient conditions while permitting enhanced RNA turnover when metabolic resources are limited.

RNA degradation is one of the processes involved in the induction of autophagy. In plants, autophagy plays a role in regulating plant growth and development and in adaptation to stress situations [31]. A central regulator of this process is Target of Rapamycin kinase (TOR), which integrates nutrient and energy signals to balance anabolic and catabolic activities [32]. Interestingly, GTP has been reported to act as an activator of TOR signaling [33], promoting biosynthetic processes (including nucleic acid biosynthesis) while repressing autophagy-dependent degradation pathways [34]. GTP would have a dual role of inhibiting ribonuclease and activating the TOR pathway, providing a mechanistic basis for the plant growth-defense trade-off. High GTP levels signal metabolic sufficiency, driving TOR-dependent growth processes while keeping PvRNS2 in check. Upon stress perception, the disruption of this nucleotide equilibrium suppresses TOR and activates PvRNS2-mediated RNA metabolism. This dual-action switch ensures that cellular resources are dynamically reallocated: suppressing energy-costly growth while fueling the nucleotide recycling necessary to sustain localized defense responses and survival.

Coinciding with the increase in RNase activity, mechanical wounding induced the expression of two S-like ribonuclease T2 genes, *PvRNS2* and *PvRNS3*, in both leaf and radicle, although with distinct temporal dynamics. *PvRNS2* showed a rapid and transient induction, consistent with its identification as the primary contributor to the observed ribonuclease activity. *PvRNS2* transcript accumulated rapidly to peak at 2 h post-treatment. However, the maximum ribonuclease activity was determined at 24 h post-treatment. This phenomenon can be attributed to the necessary time required for protein translation, post-translational modification, and subsequent enzyme accumulation and stability. However, *PvRNS3* exhibited a delayed and more modest induction. These differential expression kinetics may reflect functional specialization within the RNase T2 family, with *PvRNS2* associated with early responses requiring rapid metabolic reorganization, such as nutrient mobilization, whereas *PvRNS3* may participate in more specialized and less immediate responses, potentially related to prolonged adjustments. This interpretation is consistent with previous observations indicating that *PvRNS2* accumulates at high levels in cotyledons during developmental stages characterized by intense nutrient mobilization [18], while *PvRNS3* expression is associated with salt stress responses in radicles and with seed coat tissues [17,19]. The induction of both genes in aerial and subterranean tissues further underscores the existence of a systemic response to wounding, allowing coordinated protection of photosynthetic and root tissues against mechanical stress or pathogen attack. *PvRNS2* was the only gene that increased its expression with MeJA. The lack of response for *PvRNS3* might be because MeJA triggers a weaker induction than mechanical wounding. Specifically, wounding increased *PvRNS2* expression by 55-fold in the radicle compared to only a 2-fold increase by MeJA. Given that the wounding response of *PvRNS3* is already substantially lower than that of *PvRNS2* (3-fold versus 55-fold), its induction by MeJA may fall below detection levels. It is also important to address the lack of induction observed for *PvRNS2* expression in other stresses such as salt or water stresses.

Most research on ribonucleases T2 has been conducted in *Arabidopsis*, where five genes have been described [35]. Among them, *AtRNS1* has been shown to be induced by wounding and other stress conditions [36,37,38,39]. *PvRNS2* and *PvRNS3* are the closest homologs of *AtRNS1* in common bean, and as happened with *AtRNS1,* both genes are induced by wounding, but with different patterns, suggesting that in common bean both genes share the function of wound response attributed to *AtRNS1* in *Arabidopsis* but with specialized roles.

Nucleotides released from ribonuclease activity can be either recycled or degraded. The degradation of purine nucleotides can lead to the synthesis of ureides, molecules rich in nitrogen, which are especially relevant in ureidic legumes such as common bean. Despite the strong induction of *PvRNS2* expression, we did not observe a concomitant induction of genes encoding nucleotidase or nucleosidase involved in complete nucleotide degradation. Instead, a decrease in expression of several genes has been reported, such as *PvNTD1* and *PvNSH2*, as well as *PvALN1*. This suggests that at least a fraction of the nucleotides generated by RNA degradation may be preserved for reutilization rather than being fully degraded. In *Arabidopsis*, activation of the purine degradation pathway has been associated with the accumulation of ureides during wounding, where they act as antioxidants to limit ROS levels and to decrease tissue mortality in the damaged leaves [40]. In common bean, *PvALN1* expression was also decreased, but only transiently, suggesting differences in the regulation of purine metabolism during the wound response between these species. It is interesting that there is a decrease in expression of *PvNDPKII*, a nucleoside diphosphate kinase involved in generating NTP from ATP. In *Populus trichocarpa*, PtNDPK2 has been associated with tolerance to abiotic stresses [41]. The uncoupled transcriptional response in common bean between ribonuclease and other nucleotide-degrading genes highlights an elegant physiological strategy tailored to localized mechanical injury or herbivory. Wounding demands immediate energy-efficient metabolic reprogramming to support defense and tissue repair. Because de novo nucleotide biosynthesis is an extraordinarily energy-costly process, the total catabolism of RNA degradation products would represent a metabolic penalty. By keeping downstream degradation genes at basal expression levels, common bean prioritizes salvage pathways. The mononucleotides liberated by PvRNS2 are preferentially retained within the local cellular pool rather than being broken down for nitrogen export. This immediate availability of recycled purines and pyrimidines likely serves two critical survival functions: it fulfills the sudden transcriptomic demand for defense-related gene expression and ensures the rapid replenishment of GTP/ATP pools necessary to maintain basal homeostasis under threat.

Beyond nutrient recycling, wound-induced RNA degradation may also play signaling functions. The generation of RNA fragments could contribute to the pool of signaling molecules called damage-associated molecular patterns (DAMPs) released upon tissue disruption. Although extracellular DNA (eDNA) has been proposed to function as DAMPs in plants [42,43], whether eRNA fragments play any signaling role during wound responses remains an open question. Recent empirical findings have suggested that extracellular self-RNA (eRNA) functions as a potent DAMP that activates pattern-triggered immunity outputs, including downstream defense genes [13]. Beyond the perception of intact extracellular RNA, highly coordinated enzymatic processing is required to orchestrate the wound response. In this context, ribonucleases could serve as critical upstream processors; upon mechanical wounding, these enzymes are mobilized to degrade RNA, leading to the biogenesis of specific populations of tRNA-derived fragments and other small RNA fragments that could act as secondary molecular signals [44,45]. Wounds increase ribonuclease activity towards tRNA in common bean, raising the possibility that the resulting tRNA fragments could contribute to wound-related signaling processes, as previously suggested for AtRNS1 [39,44]. Moreover, recent studies in rice have revealed non-canonical functions of RNase T2 family members, such as OsRNS4, which lacks catalytic activity but participates in the transport of small RNAs involved in the antifungal defense to the apoplastic space [46]. Together, these findings highlight the functional versatility of plant ribonuclease T2 and underscore the need to extend mechanistic studies beyond *Arabidopsis* to agronomically relevant crops.

To summarize, our results demonstrate that mechanical wounding leads to the rapid induction of both ribonuclease activity and expression of specific S-like RNase T2 genes in common bean. The strong and early induction of *PvRNS2* points to an important role for RNA metabolism in the wound response. Further studies will be required to elucidate the precise physiological functions of *PvRNS2*, including its contribution to nutrient recycling, autophagy regulation, and defense signaling. Given the rapid and robust responsiveness of *PvRNS2* to tissue damage, this gene may also serve as a molecular marker for wound-induced signaling pathways. It will be interesting to determine the molecular components involved in the signaling cascade that induces the rapid expression of the *PvRNS2* gene, not only through molecular analyses but also through biochemical analyses and the use of different inhibitors. It will also be interesting to determine the hormones involved in the pathway. Further understanding of the RNA turnover in wound and stress responses may provide new opportunities to improve crop resilience without compromising productivity.

## 4. Materials and Methods

### 4.1. Plant Material

*Phaseolus vulgaris* seeds of the Great Northern variety were sterilized through the following treatment: immersion in ethanol for 30 s, followed by another in 0.2% (*w*/*v*) NaClO for 10 min. Once sterilized, the seeds were washed with distilled water at least six times and were placed in 150 mm-diameter Petri dishes containing three paper disks moistened with 10 mL of sterile distilled water and covered with a fourth layer of paper disk moistened with 2 mL of water. The dishes were placed in a growth chamber under a photoperiod of 16 h light/8 h dark, with a daytime temperature of 26 °C, a nighttime temperature of 20 °C, and a constant relative humidity of 70%. The photosynthetic photon flux density was 300 μmol m^−2^ s^−1^. The amount of water inside the plates was maintained by adding distilled water daily.

For plant cultivation, the seedlings were transferred to pots 6 days after the start of imbibition containing vermiculite: perlite (3:1, *v*/*v*) and placed in the growth chamber under the same conditions indicated above. The plants were watered 3 times a week with nutrient solution supplemented with 10 mM nitrate [47].

Wounded leaves were obtained by gently marking the leaves of plants at 21 days post-imbibition with serrated tweezers. Mechanical damage was realized by marking approximately 8–10 times per leaflet, leaving around 1 cm between each wound. Samples were collected at 0.5, 2, 10, and 24 h after wounding and non-treated leaves as a control.

The analysis with radicles subjected to different treatments was performed in radicles isolated from seedlings grown in Petri dishes. The radicles were always obtained from seedlings at 6 days post-imbibition. Wounded radicles were obtained as described for leaves but by using plant material for the radicles, and marking the entire length of the radicle, leaving around 3 mm between each mark. In other treatments, sterile water was supplemented with methyl jasmonate: 250 µM (MeJA), NaCl: 200 mM (NaCl), 5 mM of salycilic acid (SA) 7.5%, PEG 8000 (PEG), and only sterile water as control.

In all cases, tissue samples were collected, immediately frozen in liquid nitrogen, and stored at −80 °C until further processing.

### 4.2. Preparation of Crude Extracts

Frozen plant material was ground using a mortar and pestle under liquid nitrogen and stored at −80 °C. Crude extracts were obtained by mixing in a tube approximately 100 mg of powder with extraction buffer (50 mM TES, pH 7, and 3.5 mM sodium deoxycholate) in a 4:1 (*v*/*w*) ratio, and the homogenate was obtained with the use of a plastic swab. The homogenate was centrifuged at 14,000× *g* for 15 min at 4 °C, and the supernatant was collected in new tubes and considered the crude extract.

### 4.3. Determination of Total Protein

Protein content in the crude extracts was determined by the protein–dye binding method [48] using the Bio-Rad reactive substance and with bovine serum albumin as standard.

### 4.4. Determination of Enzymatic Activities

#### 4.4.1. In Vitro Assays

Reaction mixtures were prepared by adding 125 μL of nucleic acid solution (RNA from Torula yeast, DNA from salmon testes as dsDNA, denatured DNA from salmon testes as ssDNA, and tRNA from *Saccharomyces*) at a concentration of 2.4 mg/mL, 50 μL of BSA at 1 mg/mL, crude extract, and 50 mM acetate buffer pH 5.5 up to 600 μL. The enzymatic reactions were carried out at 40 °C for 30 min. Aliquots of 200 μL were taken from reaction mixtures before and after the reaction, and then 20 μL of ammonium acetate 7.5 M and 500 μL of ethanol were added to each tube. The tubes were placed at −80 °C overnight to precipitate nucleic acids. Afterwards, tubes were centrifuged for 15 min at 4 °C, and the absorbance at 260 nm was determined in the supernatants. One unit of enzymatic activity was defined as the amount of enzyme that catalyzed an increase of 1 unit of absorbance at 260 nm per minute.

#### 4.4.2. In-Gel Assays

Crude extracts were incubated with loading buffer (0.125 M Tris pH 6.8, 10% [*v*/*v*] glycerol, 2% [*w*/*v*] SDS, and 0.01% [*w*/*v*] bromophenol blue) and incubated at 60 °C for 10 min. Next, the samples were loaded into a polyacrylamide gel that polymerized in the presence of RNA.

For the preparation of the separating minigels, Torula yeast RNA (2 mg mL^−1^) was added during polymerization. Electrophoresis was performed at a constant 100 V and 4 °C. Subsequently, SDS was removed by two 10 min washes with 0.01 M acetate buffer (pH 5.5) supplemented with 25% isopropanol, followed by two rinses in alcohol-free buffer at 4 °C. Enzymatic incubation was generally performed at 50 °C in 0.05 M buffer for periods of 20 to 30 min. Finally, the gels were stained for nucleic acids with 0.2% toluidine blue O. The gels are shown after inverting the images.

#### 4.4.3. Ribonuclease Activity Using rRNA

Total RNA isolated from common bean radicles was used as the substrate. The enzymatic reaction was carried out in a total volume of 20 μL, adding 2 μg of RNA, an appropriate amount of crude extract, and 50 mM acetate buffer (pH 5.5). The reaction was carried out at 37 °C. Aliquots of 10 μL were taken at time 0 and 10 min after the reaction, and 0.33 μL of MOPS buffer pH 7 (200 mM MOPS, 50 mM sodium acetate, and 1 mM EDTA-Na2) and 0.6 μL of formaldehyde were added and then gently mixed. These mixtures were loaded onto an agarose gel and subjected to electrophoresis.

### 4.5. Proteomic Analysis

The portion of the gel with ribonuclease activity was diced and kept in water. Afterwards, the sample was subjected to trypsin digestion at the Proteomics Facility at Research Support Central Service (SCAI) at the University of Cordoba, as previously described [49]. The obtained peptides were analyzed by high-resolution mass spectrometry using a nanoElute nanoflow ultrahigh-pressure LC system coupled to a timsTOF Pro 2 mass spectrometer(Bruker Daltonics, Bremen, Germany), equipped with a Captive Spray nanoelectrospray ion source at SCAI (Universidad de Cordoba), as previously described [47].

### 4.6. RNA Isolation and cDNA Synthesis

Total RNA was extracted using the NZYol Reagent (NZYTECH, Lisbon, Portugal) following the manufacturer’s instructions, with the exception of an additional LiCl precipitation step included at the end to enhance RNA quality. Two micrograms of total RNA were treated with RNase-free DNase I (NEB), and the absence of contaminating genomic DNA was confirmed by PCR. First-strand cDNA synthesis was performed using DNase-treated RNA, RevertAid Reverse Transcriptase (Thermo Fisher, Waltham, MA, USA), and random hexamer primers.

### 4.7. qRT-PCR Analysis

Quantitative RT-PCR (qRT-PCR) was performed on a CFX Connect system(with software CFX Maestro 4.1.2433.1219) (Bio-Rad, Hercules, CA, USA) using the iTaq Universal SYBR Green Supermix (Bio-Rad) and the primers listed in Table S2. The amplification protocol consisted of an initial denaturation at 95 °C for 5 min, followed by 40 cycles of 15 s at 95 °C, 30 s at 60 °C, and 30 s at 72 °C. After completion of the final cycle, a melting-curve analysis was conducted from 60 to 100 °C increments in 0.5 °C to assess reaction specificity. Gene expression levels were normalized using the geometric mean of two housekeeping genes (ubiquitin and actin) and quantified using the 2^−ΔCT^ or 2^−ΔΔCT^ method [50]. Primer specificity was verified by sequencing the corresponding PCR products.

### 4.8. Statistical Analysis

All results represent the mean of four independent biological replicates, each with three technical replicates. All statistical analyses for multiple-treatment comparisons were performed using IBM SPSS Statistics (28.0.0.0(190)). Data are presented as mean ± standard error (SE). For qRT-PCR analyses, relative expression values were log10-transformed prior to statistical testing to reduce the effect of differences in magnitude among treatments and to improve variance homogeneity. Homogeneity of variances was assessed using Levene’s test.

For experiments involving more than two treatments, differences among groups were analyzed by one-way ANOVA. When the assumption of homogeneity of variances was met, Tukey’s HSD post hoc test was used for multiple comparisons. When variances were heterogeneous, Welch’s ANOVA followed by Games–Howell post hoc comparisons was applied. Differences were considered statistically significant at *p* < 0.05.

For experiments in which each treatment was compared directly with the control, Student’s two-tailed *t*-tests were performed using Microsoft Excel. Asterisks indicate significant differences relative to the control (* *p* < 0.05, ** *p* < 0.01, and *** *p* < 0.001).

## Data Availability

The original contributions presented in this study are included in the article/Supplementary Material. Further inquiries can be directed to the corresponding author.

## Supplementary Materials

The following supporting information can be downloaded at: https://www.mdpi.com/article/10.3390/plants15121907/s1; Table S1: Protein identified after proteomic analysis. Protein corresponding to PvRNS2 is marked in yellow. Table S2: Primer used in the present study.
