# Supplementary material for: Mechanical Wounding Induces Rapid RNA-Degrading Activity Mediated by the S-like Ribonuclease PvRNS2 in Common Bean
Source: Plants (Basel). 2026 Jun 19;15(12):1907. doi: 10.3390/plants15121907 (PMC13306568; doi:10.3390/plants15121907)
Supplement: Supplementary file 1 [file plants-15-01907-s001.zip › plants-4348857-supplementary.pdf]

Table S1. Protein identified after proteomic analysis. Protein corresponding to PvRNS2 is marked in yellow.

-10lgP	Coverage (%)	#Peptides	#Unique	Avg. Mass	Description
164.85	59	12	11	18174	Peptidyl-prolyl cis-trans isomerase OS=Phaseolus vulgaris OX=3885 GN=PHAVU_011G026900g PE=3 SV=1
107.51	15	4	4	26102	Thaumatin-like protein OS=Phaseolus vulgaris OX=3885 GN=PHAVU_002G155500g PE=3 SV=1
101.83	15	4	4	28809	Peptidyl-prolyl cis-trans isomerase OS=Phaseolus vulgaris OX=3885 GN=PHAVU_007G001100g PE=3 SV=1
96.62	21	4	4	22342	Uncharacterized protein OS=Phaseolus vulgaris OX=3885 GN=PHAVU_004G138500g PE=4 SV=1
95.76	14	3	3	26647	PsbP C-terminal domain-containing protein OS=Phaseolus vulgaris OX=3885 GN=PHAVU_008G015100g PE=4 SV=1
78.49	27	2	2	14056	Uncharacterized protein OS=Phaseolus vulgaris OX=3885 GN=PHAVU_007G155500g PE=3 SV=1
67.02	25	2	2	13805	Uncharacterized protein OS=Phaseolus vulgaris OX=3885 GN=PHAVU_008G133700g PE=3 SV=1
65.24	13	3	3	25613	Glutathione peroxidase OS=Phaseolus vulgaris OX=3885 GN=PHAVU_002G288700g PE=3 SV=1
64.87	17	3	3	19804	Thioredoxin domain-containing protein OS=Phaseolus vulgaris OX=3885 GN=PHAVU_005G071200g PE=4 SV=1
62.38	14	2	2	17850	60S ribosomal protein L12 OS=Phaseolus vulgaris OX=3885 GN=PHAVU_011G079400g PE=3 SV=1
62.38	12	2	2	21816	60S ribosomal protein L12 OS=Phaseolus vulgaris OX=3885 GN=PHAVU_007G220300g PE=3 SV=1
59.52	16	2	2	12480	Ubiquitin-like domain-containing protein (Fragment) OS=Phaseolus vulgaris OX=3885 GN=PHAVU_003G1236000g PE=4 SV=1
59.52	14	2	2	14579	Ubiquitin-like domain-containing protein OS=Phaseolus vulgaris OX=3885 GN=PHAVU_005G060100g PE=4 SV=1
59.52	12	2	2	17257	Ubiquitin-like domain-containing protein OS=Phaseolus vulgaris OX=3885 GN=PHAVU_005G060100g PE=4 SV=1
59.52	9	2	2	21601	Ubiquitin-like domain-containing protein (Fragment) OS=Phaseolus vulgaris OX=3885 GN=PHAVU_003G1236001g PE=4 SV=1
59.52	7	2	2	30108	Ubiquitin-like domain-containing protein (Fragment) OS=Phaseolus vulgaris OX=3885 GN=PHAVU_003G1236001g PE=4 SV=1
59.52	6	2	2	34185	Ubiquitin-like domain-containing protein OS=Phaseolus vulgaris OX=3885 GN=PHAVU_003G1236001g PE=4 SV=1
59.52	5	2	2	40194	Ubiquitin-like domain-containing protein OS=Phaseolus vulgaris OX=3885 GN=PHAVU_003G123400g PE=4 SV=1
59.52	5	2	2	42699	Ubiquitin-like domain-containing protein OS=Phaseolus vulgaris OX=3885 GN=PHAVU_007G052600g PE=4 SV=1
59.08	5	2	2	53244	6-phosphogluconate dehydrogenase, decarboxylating OS=Phaseolus vulgaris OX=3885 GN=PHAVU_008G023700g PE=3 SV=1
57.00	8	2	1	28232	Peptidyl-prolyl cis-trans isomerase OS=Phaseolus vulgaris OX=3885 GN=PHAVU_001G219400g PE=3 SV=1
55.26	8	1	1	17995	RRM domain-containing protein OS=Phaseolus vulgaris OX=3885 GN=PHAVU_009G023700g PE=4 SV=1
53.27	10	2	2	41715	Actin OS=Phaseolus vulgaris OX=3885 GN=PHAVU_005G143500g PE=3 SV=1
53.27	10	2	2	41627	Actin OS=Phaseolus vulgaris OX=3885 GN=PHAVU_011G064500g PE=2 SV=1
53.27	10	2	2	41683	Actin OS=Phaseolus vulgaris OX=3885 GN=PHAVU_001G142500g PE=3 SV=1
53.27	10	2	2	41773	Actin OS=Phaseolus vulgaris OX=3885 GN=PHAVU_008G011000g PE=3 SV=1
53.27	10	2	2	41687	Actin OS=Phaseolus vulgaris OX=3885 GN=PHAVU_007G260000g PE=3 SV=1
52.89	9	2	2	28017	Uncharacterized protein OS=Phaseolus vulgaris OX=3885 GN=PHAVU_002G170800g PE=3 SV=1
51.69	8	3	3	43035	Fructose-bisphosphate aldolase OS=Phaseolus vulgaris OX=3885 GN=PHAVU_011G039100g PE=2 SV=1
50.67	9	1	1	16586	UBC core domain-containing protein OS=Phaseolus vulgaris OX=3885 GN=PHAVU_007G270100g PE=4 SV=1
49.57	7	1	1	20895	glutathione-specific gamma-glutamylcyclotransferase OS=Phaseolus vulgaris OX=3885 GN=PHAVU_008G163900g PE=4 SV=1
49.35	9	1	1	15310	HMG box domain-containing protein OS=Phaseolus vulgaris OX=3885 GN=PHAVU_009G012000g PE=3 SV=1
49.35	9	1	1	16701	HMG box domain-containing protein OS=Phaseolus vulgaris OX=3885 GN=PHAVU_009G012000g PE=3 SV=1
49.20	6	2	2	40044	GDSL esterase/lipase OS=Phaseolus vulgaris OX=3885 GN=PHAVU_006G169100g PE=3 SV=1
48.00	8	2	2	31043	protein-serine/threonine phosphatase OS=Phaseolus vulgaris OX=3885 GN=PHAVU_005G074100g PE=3 SV=1
47.56	6	1	1	21214	Glutaredoxin-dependent peroxiredoxin OS=Phaseolus vulgaris OX=3885 GN=PHAVU_006G151800g PE=3 SV=1
45.08	12	3	3	35585	Carbonic anhydrase OS=Phaseolus vulgaris OX=3885 GN=PHAVU_004G013500g PE=3 SV=1
45.08	12	3	3	36890	Carbonic anhydrase OS=Phaseolus vulgaris OX=3885 GN=PHAVU_004G013500g PE=3 SV=1
44.71	21	1	1	8410	Dirigent protein (Fragment) OS=Phaseolus vulgaris OX=3885 GN=PHAVU_003G265100g PE=4 SV=1
44.50	6	1	1	25243	Nucleoside diphosphate kinase OS=Phaseolus vulgaris OX=3885 GN=PHAVU_003G208500g PE=3 SV=1
43.43	5	2	2	26172	Plant basic secretory protein (BSP) family protein OS=Phaseolus vulgaris OX=3885 GN=PHAVU_007G041000g PE=4 SV=1
41.69	12	1	1	11409	Histone H4 OS=Phaseolus vulgaris OX=3885 GN=PHAVU_001G169200g PE=3 SV=1
41.69	11	1	1	11766	Histone H4 (Fragment) OS=Phaseolus vulgaris OX=3885 GN=PHAVU_011G163600g PE=3 SV=1

41.69	10	1	1	14048	Histone H4 OS=Phaseolus vulgaris OX=3885 GN=PHAVU_011G164000g PE=3 SV=1
41.69	7	1	1	20839	Histone H4 (Fragment) OS=Phaseolus vulgaris OX=3885 GN=PHAVU_011G069500g PE=3 SV=1
41.69	5	1	1	28116	Histone H4 OS=Phaseolus vulgaris OX=3885 GN=PHAVU_011G164600g PE=3 SV=1
41.36	6	1	1	20506	Pectinesterase inhibitor domain-containing protein OS=Phaseolus vulgaris OX=3885 GN=PHAVU_005G007600g PE=3 SV=1
41.20	5	1	1	26325	Uncharacterized protein OS=Phaseolus vulgaris OX=3885 GN=PHAVU_009G224900g PE=3 SV=1
40.51	7	1	1	21238	chitinase OS=Phaseolus vulgaris OX=3885 GN=PHAVU_006G102200g PE=4 SV=1
40.23	8	1	1	22076	Alginate lyase 2 domain-containing protein OS=Phaseolus vulgaris OX=3885 GN=PHAVU_009G146800g PE=4 SV=1
40.05	9	1	1	16323	40S ribosomal protein S14 OS=Phaseolus vulgaris OX=3885 GN=PHAVU_006G095400g PE=3 SV=1
37.91	9	1	1	16111	ADF-H domain-containing protein OS=Phaseolus vulgaris OX=3885 GN=PHAVU_006G132700g PE=3 SV=1
37.43	8	1	1	16668	KOW domain-containing protein OS=Phaseolus vulgaris OX=3885 GN=PHAVU_006G190800g PE=3 SV=1
36.65	6	1	1	22934	Plasma membrane-associated cation-binding protein 1 OS=Phaseolus vulgaris OX=3885 GN=PHAVU_009G245700g PE=4 SV=1
35.63	7	1	1	14209	Histone H2A OS=Phaseolus vulgaris OX=3885 GN=PHAVU_009G199100g PE=3 SV=1
35.63	7	1	1	14638	Histone H2A OS=Phaseolus vulgaris OX=3885 GN=PHAVU_007G176000g PE=3 SV=1
35.63	7	1	1	14510	Histone H2A OS=Phaseolus vulgaris OX=3885 GN=PHAVU_001G155700g PE=3 SV=1
35.63	7	1	1	13951	Histone H2A OS=Phaseolus vulgaris OX=3885 GN=PHAVU_005G090400g PE=3 SV=1
35.63	7	1	1	13994	Histone H2A OS=Phaseolus vulgaris OX=3885 GN=PHAVU_005G090300g PE=2 SV=1
35.63	7	1	1	14609	Histone H2A OS=Phaseolus vulgaris OX=3885 GN=PHAVU_006G090400g PE=3 SV=1
35.63	6	1	1	14693	Histone H2A OS=Phaseolus vulgaris OX=3885 GN=PHAVU_006G095800g PE=3 SV=1
35.63	6	1	1	15207	Histone H2A OS=Phaseolus vulgaris OX=3885 GN=PHAVU_001G146100g PE=3 SV=1
35.63	6	1	1	15852	Histone H2A OS=Phaseolus vulgaris OX=3885 GN=PHAVU_005G142000g PE=3 SV=1
35.63	6	1	1	15754	Histone H2A OS=Phaseolus vulgaris OX=3885 GN=PHAVU_005G141800g PE=3 SV=1
35.63	6	1	1	15708	Histone H2A OS=Phaseolus vulgaris OX=3885 GN=PHAVU_005G141900g PE=3 SV=1
35.63	6	1	1	15966	Histone H2A OS=Phaseolus vulgaris OX=3885 GN=PHAVU_007G118400g PE=3 SV=1
35.63	6	1	1	15820	Histone H2A OS=Phaseolus vulgaris OX=3885 GN=PHAVU_005G142300g PE=3 SV=1
35.46	5	1	1	27912	Chlorophyll a-b binding protein, chloroplastic OS=Phaseolus vulgaris OX=3885 GN=PHAVU_009G186500g PE=2 SV=1
31.45	6	1	1	23131	Uncharacterized protein OS=Phaseolus vulgaris OX=3885 GN=PHAVU_006G135400g PE=3 SV=1
31.07	7	1	1	13076	Phytocyanin domain-containing protein OS=Phaseolus vulgaris OX=3885 GN=PHAVU_002G250700g PE=4 SV=1
29.94	8	1	1	17161	40S ribosomal protein S15 OS=Phaseolus vulgaris OX=3885 GN=PHAVU_007G178000g PE=3 SV=1
29.94	8	1	1	17115	40S ribosomal protein S15 OS=Phaseolus vulgaris OX=3885 GN=PHAVU_002G320200g PE=3 SV=1
29.94	8	1	1	17158	40S ribosomal protein S15 OS=Phaseolus vulgaris OX=3885 GN=PHAVU_003G089200g PE=3 SV=1
29.57	7	1	1	15406	Histone H3 OS=Phaseolus vulgaris OX=3885 GN=PHAVU_001G009100g PE=2 SV=1
27.66	10	1	1	11068	Uncharacterized protein OS=Phaseolus vulgaris OX=3885 GN=PHAVU_001G114100g PE=4 SV=1
27.50	6	1	1	27089	Triosephosphate isomerase OS=Phaseolus vulgaris OX=3885 GN=PHAVU_005G144100g PE=3 SV=1
27.44	5	1	1	24115	Uncharacterized protein OS=Phaseolus vulgaris OX=3885 GN=PHAVU_008G229600g PE=3 SV=1
27.44	5	1	1	24119	Ras-related protein RABA1f OS=Phaseolus vulgaris OX=3885 GN=PHAVU_003G073500g PE=3 SV=1
27.44	5	1	1	24211	Ras-related protein RABA1f OS=Phaseolus vulgaris OX=3885 GN=PHAVU_006G180000g PE=3 SV=1
27.44	5	1	1	24155	Ras-related protein RABA1f OS=Phaseolus vulgaris OX=3885 GN=PHAVU_011G116100g PE=3 SV=1
27.44	5	1	1	24053	Uncharacterized protein OS=Phaseolus vulgaris OX=3885 GN=PHAVU_005G081400g PE=3 SV=1
27.44	5	1	1	23778	Ras-related protein RABA2a OS=Phaseolus vulgaris OX=3885 GN=PHAVU_006G220200g PE=3 SV=1
26.96	5	1	1	17338	Glutaredoxin-dependent peroxiredoxin OS=Phaseolus vulgaris OX=3885 GN=PHAVU_004G166100g PE=3 SV=1
26.61	5	1	1	22538	Uncharacterized protein OS=Phaseolus vulgaris OX=3885 GN=PHAVU_010G048200g PE=3 SV=1
26.61	6	1	1	21553	Uncharacterized protein OS=Phaseolus vulgaris OX=3885 GN=PHAVU_005G141600g PE=3 SV=1
26.61	5	1	1	22559	Uncharacterized protein OS=Phaseolus vulgaris OX=3885 GN=PHAVU_003G224200g PE=3 SV=1
26.61	5	1	1	23664	Uncharacterized protein OS=Phaseolus vulgaris OX=3885 GN=PHAVU_011G066700g PE=3 SV=1
26.61	5	1	1	23704	Uncharacterized protein OS=Phaseolus vulgaris OX=3885 GN=PHAVU_005G141600g PE=3 SV=1

26.61	5	1	1	23915	Uncharacterized protein OS=Phaseolus vulgaris OX=3885 GN=PHAVU_008G014300g PE=3 SV=1
26.61	5	1	1	23931	Uncharacterized protein OS=Phaseolus vulgaris OX=3885 GN=PHAVU_007G011500g PE=3 SV=1
26.61	5	1	1	23907	Uncharacterized protein OS=Phaseolus vulgaris OX=3885 GN=PHAVU_007G158500g PE=3 SV=1
26.61	5	1	1	23052	Ras-related protein Rab7 OS=Phaseolus vulgaris OX=3885 GN=PHAVU_011G046100g PE=3 SV=1
26.61	5	1	1	23238	Ras-related protein Rab7 OS=Phaseolus vulgaris OX=3885 GN=PHAVU_010G149200g PE=3 SV=1
26.61	5	1	1	22352	Uncharacterized protein OS=Phaseolus vulgaris OX=3885 GN=PHAVU_006G220300g PE=3 SV=1
26.61	5	1	1	23098	Ras-related protein Rab7 OS=Phaseolus vulgaris OX=3885 GN=PHAVU_007G085200g PE=3 SV=1
26.61	5	1	1	23468	Ras-related protein Rab7 OS=Phaseolus vulgaris OX=3885 GN=PHAVU_002G265500g PE=3 SV=1
26.46	8	1	1	16730	KOW domain-containing protein OS=Phaseolus vulgaris OX=3885 GN=PHAVU_002G011200g PE=3 SV=1
25.41	7	1	1	17853	Small ribosomal subunit protein uS17 N-terminal domain-containing protein OS=Phaseolus vulgaris OX=3885 GN=PHAVU_006G060600g PE=3 SV=1
24.94	7	1	1	17263	50S ribosomal protein L23, chloroplastic OS=Phaseolus vulgaris OX=3885 GN=PHAVU_011G067600g PE=3 SV=1
24.94	6	1	1	17524	50S ribosomal protein L23, chloroplastic OS=Phaseolus vulgaris OX=3885 GN=PHAVU_008G015200g PE=3 SV=1
22.84	6	1	1	20729	60S ribosomal protein L11 OS=Phaseolus vulgaris OX=3885 GN=PHAVU_011G126600g PE=3 SV=1
22.84	6	1	1	20713	60S ribosomal protein L11 OS=Phaseolus vulgaris OX=3885 GN=PHAVU_005G011400g PE=3 SV=1

Table S2. Primer used in the present study.

Gene	Accession number	Forward 5'>3'	Reverse 5'>3'
**PVN3**	**Phvul.002G223100**	GATTGCCATGATTCTTCCAAGC	AATTCAGATGAAGGGCCTGC
**PVN4**	**Phvul.006G190700**	GGCCTGACCAAATTAGAAACT GG	ACATATCCTCCACGCCATGG
**RNS1**	**phvul.002G084500**	CTGAGCTGCCCAAGTAGCAA	CTTTGATCAAGCTCAGATTGTGC
**RNS2**	**phvul.002G170800**	CCAAGTGGTAACGGGGTCCA	GGTTGGCTCTCTGCTTCAGATC
**RNS3**	**phvul.002G084600**	GGACTCATCTGGGAACAGCC	GGGGAACACAGAGCAGTCAA
**RNS4**	**phvul.010G110200**	ATCCCCTTGGAGGCATTATC	AGCATAGGCGAAGTTCCTCA
**PvNTD1**	**Phvul.004G174200**	GAAGTGGTCGGGAATTTGTCG	ATCTCAAACCCGAATCCAATGTC
**PvNTD2**	**Phvul.011G182400**	TGAAACTCAGTGACGATGGCA AA	CTCTACCCAATCGTTGAATTTCTCA
**PvALN1**	**Phvul.006G186800**	CACATGAGTTAGTTAAATATTG ACAGGT	GGATATAATATAATACATGCAAACG CAC
**PvNSH1**	**Phvul.001G188700**	TTGGCAATGCAATGACGGAAG	GGCGTTCCACCCTTCAATGG
**PvNSH2**	**Phvul.003G000600**	TGGCAACCAAAATTTTCCTCCC	GTGACTTTCCCAGGATTAAGTTTTGC
**PvPAP26**	**Phvul.003G170500**	AAGTTGACCCGATGCTTCC	ACAGTCTCCGCTCCACTCTG
**PvNDPKI**	**Phvul.010G045400**	TGTGGAGCGTCCTTTTGCTGAG	GGCTCAGATTGAGCTGGGTT
**PvNDPKII**	**Phvul.003G208500**	AGAGATGGGAGTGCTGGGAT	TGCGTGATGTGCACTTGTTG
**PvNDPKIII**	**Phvul.002G297900**	CTGGAGCGCACTTTCATTGC	GCTGTTTGGCGAATTCCTTTG
**PvNDPKIV**	**Phvul.010G128100**	AATGGGCCCTACTGATGCAA	TGCAACCACATCTGAGGACA
**Actin**	**Phvul.006G209800.1**	GCAATTCAGGCTGTCTTGTCTT TGT	TAAATCACGGCCAGCAAGATCC
**Ubiquitin**	**Phvul.007G270100.1**	TACATGCGATCTTGGACTGGC	GGGGCTTTTCTGGGTAGTCT
